# Individual and city-level determinants of secondhand smoke exposure in China

**DOI:** 10.1186/s12942-015-0029-1

**Published:** 2015-12-29

**Authors:** Tingzhong Yang, Shuhang Jiang, Ross Barnett, Sihui Peng, Lingwei Yu

**Affiliations:** Center for Tobacco Control Research, Zhejiang University School of Medicine, Yuhangtang Road, Hangzhou, 310058 China; Department of Geography, University of Canterbury, Christchurch, 8140 New Zealand

**Keywords:** Secondhand smoke, Environmental tobacco smoke, Tobacco advertising, Anti-smoking media news coverage, China

## Abstract

**Background:**

Second hand smoke (SHS) exposure is a severe public health problem, especially in low and middle countries, but no studies have examined both individual and city-level variables influencing exposure.

**Methods:**

A cross-sectional multistage sampling design was used to survey subjects from 21 cities in China. Using a standardized questionnaire individual level information was collected. City-level variables were retrieved from the National Bureau of Statistics database. Multilevel logistic regression analysis was used to assess SHS exposure variation at both the individual and city level.

**Results:**

SHS exposure prevalence among non-smokers was 28.1 % (95 % CI 27.1–29.0). At the individual level lower educational attainment and income and higher exposure to tobacco advertising were associated with higher SHS exposure. On the other hand richer cities, and those with more anti-smoking media news coverage, had less SHS exposure. The presence of city smokefree regulations was unrelated to exposure.

**Conclusions:**

Given its human and economic costs, reducing SHS exposure should receive greater priority than it does in China. The results point to the need for the enactment of national smokefree laws in order to combat unacceptably high levels of SHS exposure.

## Background

Secondhand smoke (SHS) is also known as environmental tobacco smoke (ETS) or tobacco smoke pollution (TSP) or passive smoke, and is formed from the burning of cigarettes and other tobacco products and from smoke exhaled by the smoker [1]. Several studies have clearly linked exposure to SHS to a number of health consequences in non-smokers, including lung cancer, heart disease, and asthma in children [2, 3]. It has been estimated that approximately one-third of adults and 40 % of children and adolescents worldwide are exposed to SHS and that this exposure caused 1.0 % of all deaths and 0.7 % of the worldwide burden of disease [1, 4].

The adverse health effects of SHS have resulted in many countries banning smoking in workplaces and in indoor and outdoor public places [2, 5]. Although the initial push was to regulate the distribution of smoking in indoor public places, smoking bans are now increasingly characteristic of outdoor spaces especially around large institutions, such as hospitals and universities, as well as beaches and parks [6]. Smoking bans have been effective in reducing exposure to SHS and many studies have documented significant reductions in exposure. For example, in Britain, Jarvis and Feyerabend [7] reported a 79 % decline in children’s exposure to SHS between 1998 and 2012. While rates of exposure among children from families with manual occupations are still high, the decline in exposure was nevertheless similar to other occupational groups. Similarly in the USA, while SHS exposure for non-smokers declined from 52.5 to 25.3 % between 1999 and 2012, it still remains high especially for persons living below the poverty line (43.2 %), African Americans (46.8 %) and young children (40.6 %) [8]. However, while such inequalities remain, reductions in SHS exposure have nevertheless contributed to improved health and significant declines in hospital admissions especially for coronary events such as acute myocardial infarction [9].

Despite the implementation of the WHO Framework Convention on Tobacco Control (FCTC) many countries still lack comprehensive national smokefree legislation and exposure to SHS remains an ongoing concern. A large number of studies have documented significant social variations in SHS exposure. Most common are findings that SHS exposure is more common amongst non-smokers in poorer households where smoking rates are highest [10]. Typical of such research is that by Fischer et al. [11] who found that 46.7 % of the women in Bangladesh reported high exposure to SHS in the home with higher rates among lower income women. Similar patterns have also observed in richer countries such as the United States [12–14]. Harris et al. [13], for example, found that workplace SHS exposure was highest amongst lower income males living in rural areas, while McClure et al. [15], found that regional differences in exposure between the ‘Stroke Belt’ (states in the Deep South) and surrounding states (in the ‘Buckle of the Stroke Belt’), were most evident in the case of African Americans.

However, despite the identification of both geographic and social differences in SHS exposure little attention has been paid to identifying the relative impact of both individual and contextual factors upon exposure. This is surprising since many of the patterns identified reflect not only the concentration of smokers among more disadvantaged groups but also place effects such as the lack of enforcement of smoke free legislation. Bosdriesz et al. [16], for example, have argued that national differences in the implementation of tobacco control policies will depend upon not only national political factors, such as the presence of progressive ideologies, but also the degree of effectiveness of government agencies in enforcing such policies and of overcoming pro-tobacco lobbies. In this vein it is not surprising that in the United States and Europe SHS exposure is inversely associated with the enforcement of smokefree legislation [17, 18].

The aim of this study, therefore, is to examine determinants of SHS exposure in China paying particular attention to the importance of both individual and city-level factors. The study addresses three questions; (1) who is most likely to be exposed to SHS? (2) to what extent do city-level contextual factors, independent of individual characteristics, affect SHS exposure?; and (3) to what degree does the presence of smokefree regulations (SFRs) affect SHS exposure? These questions are particularly germane to China whose residents have relatively high exposure to SHS in the home, workplaces and public venues compared to other countries [19].

With respect to the first question we seek to examine the range of individual factors that influence SHS exposure. While individual correlates have previously been examined [20–22], these have largely focused on the influence of gender and socio-economic status [23, 24] and neglected other factors such as the influence of tobacco advertising on SHS exposure. Elsewhere studies have shown that exposure to tobacco advertising is associated with increasing prevalence of smoking [25, 26], but less attention has been devoted to the effects of advertising on SHS exposure among non-smokers. Current tobacco advertisements in China are filled with misleading and harmful information and the public is saturated by tobacco advertising across a range of milieus [27]. It is, therefore, noteworthy to explore whether smokers who reported frequent exposure to tobacco advertisements are more likely to be exposed to SHS.

Secondly we aim to explore the independent influence of contextual factors on SHS exposure. While some attention has been paid to urban–rural differences [28], city-level variations in SHS exposure, independent of individual factors, have received no attention. Mainland China is a vast territory that is characterized by cultural diversity and regions with large differences in economic and social development and tobacco control endeavors. These cultural, economic and regional diversities underscore the need for a large scale survey on SHS exposure which combines individual and contextual variables. Some researchers have provided information about the prevalence of SHS exposure at the population level, however, they provided little information about city-level factors [29, 30] or have focused on geographical variations in knowledge of the harms of SHS rather than patterns of exposure themselves [28]. Thus the present analysis includes three city-level contextual variables; urban GDP per capita as a measure of urban wealth, the presence of local smokefree regulations, and anti-smoking media news coverage. Elsewhere these factors have been found to be negatively associated with exposure to SHS [14, 18, 31, 32] and thus are explored in relation to SHS exposure in China.

Thirdly, we seek to determine whether local smokefree regulations (SFRs) also predict exposure. While this question has been addressed in US and European research it has received no attention in China. This is surprising especially given attempts by some cities and provinces to restrict smoking [33]. However, smoking in smokefree public places has neither come under strong public condemnation nor faced strong penalties, so smoking and exposure to SHS continues to be a common phenomenon in these venues [34–36]. So the question arises, therefore, of the extent to which the presence of SFRs has any effect on patterns of exposure.

In summary, the overall objective of this study is to examine the influence of these variables on SHS exposure in China. This information is needed to inform health policy, plan prevention strategies, and design and implement appropriate and targeted interventions.

## Methods

### Study area and participants

This study employed a cross-sectional multistage sampling design. In Stage 1, 21 cities, with over 1.0 million in population, were purposefully selected from across China and differentiated by regional location: Nine were located in eastern China, five in central China, and the remaining seven in the west. About 68 % of Chinese provinces were covered by the 21 cities in this study. Most of the sample cities were provincial capitals, but we included a few non-provincial capital cities because of their size (over 1.0 million population) in order to achieve better regional representation.

Stage 2 involved selection of residential districts within each city. Two residential districts were randomly selected in the main urban zone of each city, excluding new building districts and sub-districts. In Stage 3, four communities were randomly selected within each residential district. In Stage 4, a family household registration system (“hukou”) was used to randomly sample households in each community. Individuals aged 15 years and older, and who were permanent residents were identified within each household. Finally, one eligible participant was randomly selected from each family, with eligibility being determined by birthdate closest to the contact date [27].

### Data collection

A face-to-face interview was scheduled once an individual was identified and agreed to participate in the study. All interviews were conducted using a structured self-administered questionnaire. The survey was administered privately to participants in their home or a designated quiet place, such as a backyard or community park. Interviews were conducted on Saturdays, Sundays, or during the evening or other times when the participants were available. A participant was asked to fill out a survey questionnaire of approximately 30 min duration, following instruction from an interviewer. Participants were requested to resolve any omissions, as appropriate, and were given a token of appreciation (toothbrush and toothpaste, and other small gifts valued at approximately US$ 1.00) following questionnaire completion. The study was approved by the Ethics Committee at the Medical Center, Zhejiang University, and verbal consent was obtained from all participants prior to data collection. The data was collected between June and September in 2011.

### Measures

#### Dependent variable

SHS exposure was assessed through self-report. We defined a SHS exposure as non-smokers who report daily exposure to SHS for at least 15 min per day [8, 25]. The dependent variable in this study was SHS exposure, and was coded dichotomously as 1 = exposure and 0 = no exposure.

#### Individual-level independent variables

Sociodemographic characteristics such as age, gender, ethnicity, educational level, occupation, and personal income per capita were included. Personal income was obtained by asking respondents to report the average income per person in the household in the prior year.

Exposure to tobacco advertising: respondents were asked whether they had seen any tobacco advertisements in the last 6 months (never/seldom/sometimes/often/almost always) with the responses recoded as 1 = never/seldom and 2 = sometimes/often/almost always [27].

#### City-level independent variables

The extent to which people are exposed to SHS will also reflect city-level characteristics. Four measures were used; regional urban location, city GDP per capita, anti-smoking media news coverage and the presence of smokefree regulations. Regional location was defined in terms of whether a city was located in eastern, central, and western China. The level of urban economic development [per capita Gross Domestic Product (GDP) in Yuan] was categorized into three levels: <40,000, 40,000–49,999, and 50,000+. The data were obtained from the National Bureau of Statistics [37]. The third variable, anti-smoking media news coverage, was estimated by determining the frequency of news per million population and was categorized into three levels; <20, 20–29, and 30+. The news frequency was obtained by searching the Chinese research engine “Baidu” using four key words, “smoking” or “tobacco control” or “smoking banning” or “smoking cessation” under “city name”. Each news report identified was scrutinized to ensure that it related to tobacco control, and all without tobacco control related content were excluded [38]. Finally the presence or absence of smoke-free regulations was obtained from the SFR press release for each of the 21 cities in the sample.

### Data analysis

All data were entered into a database using Microsoft Excel. The dataset was then imported into SAS (9.3 version) for the statistical analyses. Descriptive statistics were calculated for SHS exposure prevalence. Chi square analyses were conducted in determining individual and regional-variable differences in SHS exposure. These associations were confirmed employing a multilevel logistic regression model using the SAS NLMIXED procedure [40]. Series models were built for each primary predictor, with adjustment for the influence of potentially confounding variables.

We built two models. The first was the ‘null’ model, a two-level model with random intercepts. The constant was the sole predictor in accounting for cross-city variation in SHS exposure. In this base model, we entered all individual and regional level variables, as fixed main effects, to evaluate the separate impact of all individual-level and regional-level variables on SHS exposure. The independent variables in this analysis were those emerging as statistically significant (significant level: 0.05) in the Chi square tests. All categorical variables are listed in Table 1. The first category in each variable served as the referent in the analysis. Model fitting was assessed by the likelihood of a change in the −2 log. Significance of the random parameter variance estimates was assessed using the Wald joint* X*^2^ test statistic [39].

**Table 1:** Demographic characteristics of the sample and SHS exposure prevalence

Group	N	% Sample	SHS exposure prevalence	Rao–Scott Chi square and *p* value
Individual level				
Age (years)				4.31; 0.3660
<25	2110	15.1	30.6	
25–34	3061	25.5	27.7	
35–44	2113	20.8	30.0	
45–54	2044	19.8	29.0	
55+	1878	18.8	24.8	
Gender				*10.28*; *0.0013*
Male	3747	28.6	50.0	
Female	7459	71.4	19.2	
Ethnicity				*5.54*; *0.0186*
Han	9776	89.7	30.0	
Other	1430	10.3	11.0	
Education				*18.06*; *0.0004*
Elementary school or less	634	2.4	44.7	
Junior high school	2390	18.3	50.9	
High school	4089	37.4	27.2	
Junior college or more	4093	39.0	16.0	
Occupation				*40.65*; *0.0001*
Managers and clerks	561	2.6	46.0	
Professionals	586	4.9	42.7	
Commerce and service	1255	10.5	53.5	
Operations	1604	13.3	47.8	
Students	1214	9.3	43.1	
Retired	1841	24.9	14.6	
Other	4145	34.4	14.7	
Per capita income (Yuan)				*47.44*; *0.0001*
<10,000	1416	43.2		
10,000–19,999	3130	42.9		
20,000–29,999	3103	29.1		
30,000–39,999	1413	10.5		
40,000–49,999	990	9.5		
50,000+	1154	15.4		
Exposure to tobacco advertising				*12.31*; *0.0004*
No	6561	55.9	16.2	
Yes	4644	44.1	43.2	
City-level				
Regional location				3.88; 0.1433
East	4555	51.6	22.1	
Central	1666	9.9	25.3	
West	4985	38.6	36.8	
GDP per capita				*6.96*; *0.0307*
<40,000	3205	10.2	46.7	
40,000–49,999	3560	42.6	33.3	
50,000+	4441	47.2	19.2	
Anti-smoking media news coverage				*17.75*; *0.0001*
<20	8394	65.1	32.7	
20–29	942	12.2	44.7	
30 and over	1870	22.7	14.3	
Local smokefree policy				0.91; 0.3404
Yes	7562	80.6	29.5	
No	3644	19.4	21.9	

Significant *p* values are in italics

All analyses were weighted [40]. Weights included (1) sampling weights, as the inverse of the probability of selection, calculated at region, city, district, and community. (2) Non-response weights consisted of household and individual aspects. (3) Post-stratification weights were calculated using the combination of sex (male, female) and age (<25 years, 25–, 35, 45, 55 and more), based on estimated distributions of these characteristics from a national survey [41]. The final overall weights were computed as the product of the above three weights.

Chi square analyses were weighted using the overall participant-level weights, and the multilevel analysis was weighted using sampling weight in city level and subject-level weights with non-participation and post-stratification weights, respectively [29]. As there is no weight statement available for the NLMIXED procedure, these analyses were weighted though a macro method in this study [40].

## Results

A total of 18,875 individuals were identified as potential subjects for this study, among whom, 17,124 were effectively contacted and agreed to participate in the survey. Of the 17,124 surveys, 16,866 (98.5 %) were valid questionnaires and utilized in this study. The study sample included 11,206 people who were non-smokers. Subsequent analyses focused solely on this group, of which 28.1 % (95 % CI 27.1–29.0) were subject to SHS exposure.

At the individual level exposure to SHS was significantly associated with gender, ethnicity, education, occupation, income, and exposure to tobacco advertising (Table 1). Exposure was highest amongst males (50.0 %), persons of Han ethnicity (30.0 %), those engaged in commercial and service activities (53.5 %) and those earning less than 10,000 Yuan (43.2 %). People exposed to tobacco advertising also had high rates of exposure (43.2 %) compared to those who were not exposed. Age was not significantly related to exposure although young people (<25) had slightly elevated rates.

At the city level SHS exposure was significantly positively related to GDP per capita and negatively related to anti-smoking media news coverage. SHS exposure was highest in cities with the lowest GDP per capita (46.7 %) and in cities where anti-smoking media news coverage was least (32.7 %). SHS exposure showed no relationship with city regional location and nor was it related to the presence of local smokefree policies. Interestingly cities with smokefree policies had slightly higher rates of SHS exposure compared to those which has no such policies (29.5 vs 21.9 %). This was true for all income groups except one (10,000–19,999 Yuan) indicating that the presence of smokefree policies did little to alter income inequalities in SHS exposure.

The results of the multi-level analysis indicate that among the individual variables gender, education, occupation, per capita income, and exposure to tobacco advertising were associated with high rates of SHS exposure. Of the city-level indicators only urban GDP per capita and regional anti-smoking media news coverage were significantly associated with exposure (Table 2).

**Table 2:** Results of multi level analyses

Group	Null model	Full model (OR < 95 % CI)
Individual		
Gender		
Male		1.00
Female		0.45 (0.23, 0.88)**
Education		
Elementary school or less		1.00
Junior high school		1.14 (0.64,2.04)
High school		0.60 (0.31,1.14)
Junior college or more		0.35 (0.18, 0.69)**
Occupation		
Managers and clerks		1.00
Professionals		0.95 (0.53,1.70)
Commerce and service		1.20 (0.63,2.30)
Operations		0.79 (0.42,1.51)
Students		0.32 (0.16, 0.65)**
Retired		0.28 (0.11, 0.73)**
Other		0.25 (0.08, 0.72)**
Per capita income (Yuan)		
<10,000		1.00
10,000–19,999		0.80 (0.54,1.27)
20,000–29,999		0.55 (0.35, 0.87)**
30,000–39,999		0.24 (0.09, 0.64)**
40,000–49,999		0.23 (0.10, 0.55)**
50,000+		0.34 (0.14, 0.79)**
Exposure to tobacco advertising		
No		1.00
Yes		2.83 (1.23, 6.48)**
City-level		
GDP per capita		
<40,000		1.00
40,000–49,999		1.02 (0.39,3.17)
50,000+		0.39 (0.16, 0.92)*
Anti-smoking media news coverage		
<20		1.00
20–29		1.33 (0.42,4.24)
30 and over		0.26 (0.09, 0.79)**
Random parameters between regions	3.12	2.25*
Fixed parameters	2.92**	2.43*

Significant *p* values are in italics

* p < 0.05; ** p < 0.01

Significant cross-level interactions occurred between city-level GDP per capita and individual advertising exposure and between regional anti-smoking media coverage and individual advertising exposure. Cities with higher GDP per capita tended to have lower levels of exposure to tobacco advertising, both being related to lower rates of SHS exposure. The influence of tobacco advertising on SHS exposure was also weaker in cities with stronger anti-smoking media coverage; the respective ORs being 2.94 (95 % CI 1.15, 7.53), 2.61 (CI 1.28, 3.94) and 2.04 (CI 1.91, 2.18) for cities with low, middle and high rates of anti-smoking media campaigns.

## Discussion

### Key findings

This study is the first to examine both individual and regional variation in SHS exposure both in China and elsewhere. We found a SHS exposure prevalence of 28.1 % (95 % CI 27.1–29.0) among non-smokers in the study confirming the fact that SHS exposure among non-smokers is common in China.

With respect to the three aims of the study the key findings are as follows. The first aim of the study addressed the question of who is most likely to be affected by SHS. In common with other studies internationally and in China [42], this study provides additional evidence that lower income and less educated persons are more likely to be exposed to higher levels of SHS. It has been suggested that those with lower education levels have fewer opportunities and lower health awareness and are less likely to protect themselves from the harmful effects of SHS [30]. We also found that, once other variables were controlled for, that SHS exposure for students and retired people was lower than for managers and clerks. While the reasons for this among students are unclear, it may be because schools and higher educational institutions are more likely to have implemented, and enforced, smokefree policies compared to other types of public places. However, it should be mentioned that 43.1 % of students exposed to SHS still is a severe a public health question. Less exposure on the part of retired people most likely reflects the fact that they are not working or frequenting public places where smoking is common.

Our findings showed, not surprisingly, that SHS exposure was higher in males than females (50 vs 19 %) and is consistent with findings elsewhere [13]. This differs from the findings of Wang et al. [29], where higher exposure occurred amongst females. However, their study was limited to SHS exposure in households only. The high exposure among males is related to Chinese social norms and the high prevalence of smoking among the male population in China. The very low prevalence of smoking among women in China (2.4 %) compared to their male counterparts (52.9 %) [43], underscores the vulnerability of women to SHS exposure from males. In addition, males usually socialize with other male friends or colleagues, many of whom are smokers, increasing their risk to additional SHS exposure [44]. Similar, gender-based disparities are reported in other studies in other populations [22, 45]. Consistent with the findings from other studies [25, 26, 46], our findings also showed that exposure to tobacco advertising was associated with increasing prevalence of SHS exposure. Again this is unsurprising since a large literature has documented links between advertising and smoking prevalence and SHS exposure [47].

With respect to the second objective, it is evident that city-level contextual factors also had some influence above and beyond individual characteristics, on levels of SHS exposure. We found that poorer cities, independent of individual SES, had higher rates of exposure. This may reflect a greater tolerance of smoking and fewer resources available for anti-smoking programmes, compared to richer cities, with stronger anti-smoking norms, which are more likely to have enforced smokefree policies. Also important was the relationship between anti-smoking news media coverage at the city-level and individual SHS exposure. While this has been observed elsewhere [31], this study is the first to consider the influence of city-level media coverage on individual SHS exposure in a multi-level framework. We also provide new evidence about the association between anti-smoking news media coverage, tobacco advertising and SHS exposure. Where more negative media coverage occurred at the city level individual non-smokers were less exposed to tobacco advertising and consequently less SHS. From this perspective regional anti-smoking news media coverage is important in preventing SHS pollution.

Finally, it was interesting that the presence of local smokefree policies were not associated with SHS exposure. Unlike the situation in Europe [18] this suggests that the impact of local SFRs is very limited. This is consistent with other research which indicates that while many low and middle income countries have, as a result of the WHO FCTC, introduced smoking bans, they remain unenforced [48]. In China there are numerous problems in local SFRs; they tend to apply to limited public venues, lack specific enforcement guidelines, and evoke weak penalties that do not serve as a deterrent [49, 50]. Thus the introduction of a local SFR is no guarantee that SHS exposure will be reduced.

There were a number of limitations of this study. First, its cross-sectional design precludes the ability to draw causal inferences. More longitudinal studies, along the lines of Fong et al. [35], are necessary in order to explore the effectiveness of public and workplace smoking bans on the prevalence of SHS exposure for different social groups. It would be interesting to determine the extent of regulation and how this has impacted different social groups in different spatial locales. For example, Ye et al. [51] have shown that SHS exposure declined more significantly in places with more comprehensive smoking bans but provided no disaggregation of the population groups who benefited most from such changes. Second, given that only urban residents were included in the survey the results are not generalizable to the overall population of China, which has a very substantial rural component. It is evident that, both in China [28] and elsewhere [12], that SHS exposure is particularly high in rural areas [29]. Exploring the reasons for these differences as well as between different geographic regions would seem to be a high priority. However, because our sample included 21 cities it provides a good regional coverage of urban trends in SES exposure in China. Third, although significant gender differences existed in exposure, given the patriarchal nature of Chinese society more attention is needed to the effects of male smoking particularly upon lower income women and their families. More qualitative research along the lines of Mao et al. [52] would seem to be a high priority. Finally, given that the presence of local SFRs was unrelated to SHS exposure begs the question of under what circumstances are local SFRs likely to be more effective in reducing exposure? Although examining this question was beyond the reach of this study it is an important one especially given the presence of socio-economic inequalities in SHS exposure and the heavy economic burden this has produced in China [53].

### Implications

This study provides new information about individual and city-level predictors of SHS exposure in urban China. The main message is that SHS exposure is not just a problem of individuals but also has to be set in its environmental context, whether this be a poorer city with strong smoking norms and ineffective smokefree regulations or a rural area where knowledge of the harmful effects of SHS is poor. Changes in SHS exposure can only come about through the effective implementation of national or provincial policy changes and cannot just be seen as a reflection of individual responsibility. While certain cities have recently recorded declines in SHS exposure these have been modest and exposure rates remain unacceptably high [35]. Tackling such problems thus requires effective enforcement of smokefree legislation at the citywide scale and not cosmetic changes in policy which are insufficient to deal with the poor health [54] and poor economic outcomes [55] which smoking and SHS exposure produce.

In common with many other middle income and poorer countries, overall public awareness of the hazards of passive smoking in China is low and this lack of awareness is also an important factor in SHS exposure. Mass media is a powerful way to provide information about the health effects of SHS to the general public and the city-level findings of this study tend to support this view. Therefore, the key challenge for China is to strengthened efforts to take advantage of mass media to extend the reach of tobacco control messages, especially when they also help lead to a reduction in the effects of tobacco advertising on smoking and exposure to SHS. Already there is some indication this is occurring. In 2015 the National People’s Congress in China introduced a new advertising law placing greater restrictions on tobacco advertising [56]. Efforts to restrict SHS exposure smoking should also simultaneously implement public education campaigns. Mass media is powerful way to extensively disseminate information about the health effects of SHS to the general public, and efforts should be strengthened to take advantage of mass media to extend the reach of tobacco control messages.

## Conclusion

If China wishes to be seen to adhering to the principles of the FCTC then the introduction of national smokefree bans, both in workplaces and public venues, along with stricter controls on tobacco advertising, is a high priority. Such actions are not just necessary from an economic and health point of view but are also important if China wishes to be seen as a responsible member of the international community and places a greater emphasis on protecting the health of its citizens.
